# Accuracy of Administratively-Assigned Ancestry for Diverse Populations in an Electronic Medical Record-Linked Biobank

**DOI:** 10.1371/journal.pone.0099161

**Published:** 2014-06-04

**Authors:** Jacob B. Hall, Logan Dumitrescu, Holli H. Dilks, Dana C. Crawford, William S. Bush

**Affiliations:** 1 Center for Human Genetics Research, Vanderbilt University, Nashville, Tennessee, United States of America; 2 Vanderbilt Technologies for Advanced Genomics (VANTAGE), Vanderbilt University, Nashville, Tennessee, United States of America; University of Louisville, United States of America

## Abstract

Recently, the development of biobanks linked to electronic medical records has presented new opportunities for genetic and epidemiological research. Studies based on these resources, however, present unique challenges, including the accurate assignment of individual-level population ancestry. In this work we examine the accuracy of administratively-assigned race in diverse populations by comparing assigned races to genetically-defined ancestry estimates. Using 220 ancestry informative markers, we generated principal components for patients in our dataset, which were used to cluster patients into groups based on genetic ancestry. Consistent with other studies, we find a strong overall agreement (Kappa  = 0.872) between genetic ancestry and assigned race, with higher rates of agreement for African-descent and European-descent assignments, and reduced agreement for Hispanic, East Asian-descent, and South Asian-descent assignments. These results suggest caution when selecting study samples of non-African and non-European backgrounds when administratively-assigned race from biobanks is used.

## Introduction

Hospital-based biobanks linked to electronic medical records (EMRs) are a growing and cost-effective way to ascertain large segments of a population for biomedical research studies. Genetic and clinical studies increasingly require larger numbers of samples to provide statistical power to discover genetic variation associated with complex human diseases; using existing surveyed clinical populations is a way to meet this demand quickly. Multiple studies have been published illustrating the basic utility of biobanks for validating existing association studies [1], performing phenome-wide association studies [2], [3], and for identifying novel genetic associations within existing genotype-phenotype databases [4]. The use of EMR-based biobanks for research purposes is expected to grow in the coming years [5], [6].

The Vanderbilt DNA biobank (BioVU) contains nearly 160,000 DNA samples linked to electronic medical records at Vanderbilt University and continues to accrue additional patient samples. DNA is extracted from discarded blood samples collected during routine patient care. EMR data is drawn from administrative databases and scrubbed of identifying information to generate a resource for researchers known as the Synthetic Derivative (SD) [1], [7]. A subset of the SD population has linked DNA samples, forming the BioVU subset. Upon institutional approval of a BioVU project, samples with the phenotype of interest, based on data from the SD, can be accessed and genotyped. All genotype data generated using BioVU samples is then made available to Vanderbilt investigators for future studies. The BioVU design has the distinct advantage of rapid sample accrual for a variety of clinical traits present in the patient population; however, re-contacting participants for sample collection or validation of subject data is prohibited by both institutional policy and the de-identification process, limiting some applications of the data.

With increased emphasis on the use of DNA biobanks, it is important to note the critical role of race in genetic association studies. A sample drawn from multiple underlying populations is subject to population stratification, where each population has a slightly different genetic architecture. If not properly accounted for, these differences in allele frequency can result in false associations. As such, it is common practice in genetic studies to correct for underlying population sub-structure by estimating global genetic ancestry for each sample [8]. This is often accomplished by genotyping a set of ancestry informative markers (AIMs) which are evaluated using either principal components analysis (for a continuous estimate of ancestry group) [9] or cluster analysis (for a categorical ancestry assignment) [10]. The individual measure of genetic ancestry is then used to stratify individuals or to include them as a covariate for adjustment in statistical analyses to avoid confounding.

In lieu of genotyping AIMs, genetic studies sometimes use self-reported race as a covariate, either as a surrogate for genetic ancestry or to capture social and demographic components [11]. The complex nature of the relationship between race and genetic ancestry has been extensively explored [12], and multiple studies have shown that self-reported race is generally reflective of an individual's genetic ancestry but does not account for population substructure [13], [14]. While self-reported race is commonly collected in epidemiologic cohorts, many provider-based studies use third-party reported race rather than self-reported race. Studies of agreement between self and third-party race assignment have been conducted, but have conflicting results, showing varying levels of agreement[13]–[16].

Dumitrescu et al. [17] previously reported on the utility of using third-party reported race for African-descent and European-descent individuals within BioVU, citing a high concordance with genetic ancestry. However, third-party assignment of these racial categories may be influenced by subjective criteria for specific racial groups. This notion is supported by a study that reported high accuracy for distinguishing African American and European American individuals (positive predictive value 0.95 & 0.94, respectively) using third-party reporting, but less accuracy for Hispanics and American Indians (positive predictive value 0.81 & 0.50, respectively)[18].

The accuracy of third-party racial assignments is especially critical for biobank-based studies. Should an investigator seek to perform a genetic study within a diverse population, sample selection is likely dependent on the third-party racial assignment within the EMR. As a result, samples of a different ethnicity may be selected and genotyped, only to be excluded from analysis after ancestry is determined using genetic data, resulting in a waste of research funds. Additionally, genetic ancestry can influence some clinical decision-making processes, including automated decision support, which is being integrated into some EMRs [19], [20]. Before decision support rules are implemented that consider race in treatment decisions, it is important to characterize the accuracy of race within EMRs. In this work, we characterize how well administrative third-party race assignment within BioVU reflects ancestry estimated from genetic data.

## Methods

### Ethics Statement

BioVU, Vanderbilt University's biobank, uses de-identified patient electronic medical records. This study is considered non-human subjects research by the Vanderbilt institutional review board.

### Sample Selection

A total of 7,252 individuals were selected from BioVU, specifically to over-represent diverse populations and individuals with “unknown” administrative race assignments. Within the synthetic derivative (SD) and BioVU, race is administratively assigned to one of eight predefined categories: White (W), Black (B), Asian/Pacific (A), Native American (N), Indian (I), Hispanic (H), other (O), or unknown (U) (Table 1). Based on communications with clinical personnel who regularly assign race codes, in practice, the Native American (American Indian) and Indian (South Asian) race codes are sometimes incorrectly used interchangeably. No individuals with “other” ethnicity were selected in this study. For this paper, we will refer to the predefined, administratively-assigned racial categories as Caucasian, African American, Asian/Pacific, Native American, Indian, and Hispanic (Table S1).

**Table 1:** Distribution of administratively-assigned race.

Race	Study Sample	BioVU	Synthetic Derivative	Davidson Co.*
Caucasian	4,232 (58.4%)	102,018 (64.4%)	1,116,837 (51.6%)	385,039 (61.4%)
African American	1,094 (15.1%)	14,223 (9.0%)	191,246 (8.8%)	173,730 (27.7%)
Asian/Pacific	228 (3.1%)	1,380 (0.9%)	14,449 (0.7%)	15,083 (2.4%)
Hispanic	230 (3.2%)	2,147 (1.3%)	37,466 (1.7%)	**
Native American	184 (2.5%)	212 (0.1%)	1,868 (0.1%)	2,091 (0.3%)
Indian	7 (0.1%)	1,711 (1.1%)	20,613 (1.0%)	4,338 (0.7%)
Unknown	1,277 (17.6%)	36,696 (23.2%)	781,074 (36.1%)	46,400 (7.5%)
**Total**	**7**,**252 (100%)**	**158**,**387 (100%)**	**2**,**163**,**553 (100%)**	**626**,**681 (100%)**

Race categories listed are based on classification options originating from the SD. Our BioVU dataset contained no individuals labeled Other (O). Vanderbilt University Medical Center is located in Davidson County, TN. 2010 US census data is shown for Davidson County, Tennessee [25]. * For Davidson County, “Asian/Pacific” includes Asian (Non-Indian), Native Hawaiian, and Pacific Islander individuals, “Native American” includes Native American (American Indian) and Alaskan Native individuals, “Indian” includes Asian Indian individuals, and “Unknown” includes ‘some other race’ and individuals who reported two or more races for the census. ** “Hispanic” is not listed a race in the US Census; rather, Hispanic-origin is indicated and is not exclusive to any racial category. For example, 25,156 individuals in Davidson County who self-identified as ‘White’ also self-identified, separately, as Hispanic. Within Davidson County, 9.8% of individuals indicated Hispanic origin.

### Genotyping

All 7,252 BioVU samples were genotyped using the Illumina VeraCode GoldenGate assay in the Center for Human Genetics Research (CHGR) DNA Resources Core at Vanderbilt University for 308 ancestry informative markers (AIMs) and scanned on the Illumina BeadXpress reader. AIMs genotypes were merged with existing data for 805 individuals from the International HapMap Project (Phase 3, Revision3, Build 36), including 165 CEU, 203 YRI, 137 CHB, 113 JPT, 101 GIH samples, and 86 MXL, as reference populations to assist in determining genetic ancestry (Table S1). The genetic data underwent quality control measures, including removal of 39 non-autosomal SNPs, 38 SNPs not also in the HapMap dataset, and 11 SNPs that were co-linear with principal component (PC) three and caused atypical clustering, leaving 220 SNPs for analysis (SNP list available upon request). Within the final merged dataset of 220 SNPs for 8,057 individuals, all SNPs had a minor allele frequency (MAF) greater than five percent. Of the BioVU samples in our dataset, 52% (4,192) were female.

### Genetic Ancestry Assignment

We performed principal components analysis (PCA) for 220 SNPs using the EIGENSTRAT package [9] on the combined samples. Outlier removal was disabled for all EIGENSTRAT analyses. Consistent with published studies [9], we generated the top ten principal components to estimate genetic ancestry based on genetic sharing of SNPs with HapMap samples of known continental origin. To assign genetic ancestry for each individual we performed model-based clustering, using the *mclust* [21] R package, to define and assign individuals to clusters using an ellipsoidal model with varying volume, shape, and orientation. We indicated that *mclust* should define five clusters in order to differentiate the five ancestry groups known to be present in the dataset (European-descent, African-descent, East Asian-descent, South Asian-descent, and Hispanic-descent). By plotting a 10 by 10 matrix of all pairs of PCs, colored by the defined clusters, we visually determined that PCs 1, 2, 3, 7, 9, and 10 optimally captured separation of the five clusters. These six PCs were used to perform clustering. Genetic variance within the European-descent cluster was captured in the unused principal components, and may reflect a bias toward European-descent components within this set of AIMs.

### Statistical Methods

Administratively-assigned race was compared to cluster-based ancestry assignment (Table 2) through contingency table analysis using STATA 12. Additionally, comparisons for HapMap cluster assignment is shown in Table S2. Agreement between these two classification methods was measured by Cohen's Kappa coefficient [22], which takes into account the expected agreement of two ‘raters’ based on the distribution of categories within the dataset. In this context, administrative assignment is the first ‘rater’ and genetically determined ancestry is the second ‘rater’. Kappa is standardized on a scale from -1 to 1, where 1 indicates perfect agreement, 0 indicates agreement that would be expected by chance, and negative values indicate less agreement that would be expected by chance. Genetic ancestry categories are mutually exclusive, so an individual can only be assigned to one category, based on clustering from principal components analysis.

**Table 2:** Percentages of each administratively-assigned race assigned to each genetic ancestry group.

		Genetic Ancestry				
		European	African	East Asian	Hispanic	South Asian
**Administratively-Assigned Race**	Caucasian	4,174	24	8	16	10
		(98.6%)	(0.6%)	(0.2%)	(0.4%)	(0.2%)
	African American	11	1,080	0	3	0
		(1.0%)	(98.7%)	(0.0%)	(0.3%)	(0.0%)
	Asian/Pacific	9	0	182	2	35
		(3.9%)	(0.0%)	(79.8%)	(0.9%)	(15.4%)
	Hispanic	58	8	2	154	8
		(25.2%)	(3.5%)	(0.8%)	(67.0%)	(3.5%)
	Native American	90	17	18	18	41
		(48.9%)	(9.2%)	(9.8%)	(9.8%)	(22.3%)
	Indian	3	2	0	0	2
		(42.8%)	(28.6%)	(0.0%)	(0.0%)	(28.6%)
	Unknown	1,126	83	26	21	21
		(88.3%)	(6.5%)	(2.0%)	(1.6%)	(1.6%)

Percentages reflect the proportion of individuals assigned to a genetic ancestry cluster for given administratively-assigned race.

## Results

**Figure 1:**
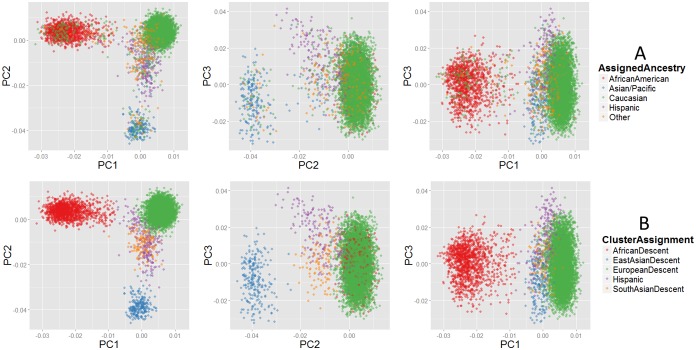
Comparison of administratively-assigned race and genetic ancestry, based on principal component analysis. A) All pairwise combinations of principle components (PCs) 1 through 3, by administratively assigned race. B) All pairwise combinations of PCs 1 through 3, by cluster assignments corresponding to genetic ancestry. Comparison of Frames 1A and1B indicate individuals with administratively assigned race different than their genetically defined ancestry cluster. For example, the East Asian-descent cluster (1B; blue) contains individuals with administratively-assigned race (1A) of Caucasian (green), Hispanic (purple), and Other (orange).

The distribution of administratively-assigned race across the sample used in this study, within BioVU, and within the entire synthetic derivative (SD)—as well as population-level counts for Davidson County Tennessee—are shown in Table 2. Plotting PC 1 versus PC 2 (Figure 1A) shows differentiation between Caucasian, African American, and Asian/Pacific assigned individuals, with Hispanic, Native American, and Indian assigned individuals falling between the three foci. Results from the model-based clustering are shown in Figure 1B. Clusters for European-descent, African-descent, and East Asian-descent clusters are distinct. The South Asian-descent and Hispanic-descent clusters are less defined, due to their varying degrees of admixture. Our ability to make inferences about the accuracy of Native American and Indian codes is limited due to ambiguous use of these codes in clinical practice, limited availability of Native American HapMap reference populations, and small sample size within our dataset. Kappa (Κ) measures of agreement between third-party race assignment and estimated genetic ancestry are shown in Table 3 (more detailed information on Kappa statistics shown in Table S3). Over the entire dataset, agreement was reasonably high (Κ = 0.872), largely driven by European-descent (Κ = 0.906) and African-descent (Κ = 0.964) individuals. Less agreement was seen for East Asian-descent (Κ = 0.825) and Hispanic-descent (Κ = 0.718) individuals. We also assessed agreement between individuals with Native American (N) and Indian (I) racial codes and South Asian ancestry estimated by the Gujarati Indian reference samples (GIH) to examine the hypothesis that these codes predominantly represent South-Asian ancestry. This agreement (Κ = 0.284) was expectedly low, indicating that while they may be misappropriated in the clinical environment, it is not strongly in favor of South-Asian ancestry. Notably, when stratifying by sex, we observe similar Kappa agreement values for European and African-descent genetic ancestry groups. In other groups, females tend to have slightly higher Kappa values than males, with the largest difference in agreement by sex observed for individuals in the South Asian-descent genetic cluster. In addition to using Kappa statistics to measure agreement, agreement can be visualized as the percent of individuals with a given administratively-assigned race assigned to each of the five genetic ancestry clustering groups (Table 2). We also examined the genetic ancestry of individuals with race status “unknown” to determine if some groups were more likely to be assigned this status than others (Table S4). The majority (88.2%) of samples with “unknown” race are genetically of European-descent, consistent with the overall representation of European-descent individuals in BioVU. African-descent individuals constitute 6.5% of the “unknown” individuals, while East Asian-descent, South Asian-descent, and Hispanic-descent individuals, each, constitute about 2%.

**Table 3:** Agreement between genetic and assigned ancestry.

Genetic Ancestry	Overall	Male	Female
Overall	0.872 (0.009)	0.862 (0.015)	0.876 (0.012)
European-descent	0.906 (0.013)	0.906 (0.020)	0.904 (0.017)
African-descent	0.964 (0.013)	0.970 (0.020)	0.960 (0.017)
East Asian-descent	0.825 (0.013)	0.800 (0.020)	0.836 (0.017)
Hispanic-descent	0.718 (0.013)	0.683 (0.020)	0.738 (0.017)
South Asian-descent	0.284 (0.012)	0.237 (0.018)	0.318 (0.016)

Notation: Cohen's Kappa coefficient (standard error).

South Asian-descent includes individuals with Native American and Indian race codes in BioVU.

Samples with administratively-assigned race of “Unknown” were excluded from this analysis.

## Discussion

Genetic and epidemiological studies routinely use self-reported race or genetic ancestry to adjust for confounding factors and/or to tailor genetic effects to specific population subgroups. Global genetic ancestry is often used to correct for population stratification in genetic analyses, because it roughly reflects differences in allele frequencies between continental populations. The social construct of race is often used to capture other demographic factors, such as access to care, dietary and environmental exposures, and socioeconomic status. Self-reported race has been shown to be highly correlated to genetic ancestry and is often used as a surrogate for continental ancestry. In many clinical datasets, self-reported ancestry is not available and various administrative procedures are used to assign race status. While it is unknown to what degree administratively-assigned race captures the various social and cultural aspects of an individual, in this work we show that it has only moderate agreement with genetic ancestry for certain populations. We observed strong agreement between administrative race assignment and genetically determined ancestry for European-descent and African-descent individuals; there was less agreement between assigned race and genetic ancestry for East Asian-descent, South Asian-descent, and Hispanic-descent individuals. Given this fact, investigators should use caution when using administratively-assigned race as a proxy for genetic ancestry, and expect some misappropriation of racial categories by third party assignment.

Interestingly, East Asian-descent, South Asian-descent, and Hispanic-descent individuals all have slightly different agreement statistics by sex, with females tending to have slightly higher agreement between administrative assignment and genetic ancestry. Previous studies have reported subjective misclassification of Hispanic individuals by sex, causing non-Hispanic females to be classified as Hispanic because of adopted spousal surnames [23]. In our data the agreement is biased slightly in the opposite direction, with females having more accurate administratively-assigned race, based on genetic ancestry estimates. While somewhat unexpected, this could be because third-party assigners are more comfortable asking females, rather than males, questions about their race and ethnicity [24].

Approximately 18% of the individuals in our dataset had an administratively-assigned race specified as “unknown” (Table 1). The distribution of genetic ancestries within these samples was significantly different from the larger dataset, with more European-descent individuals than expected (results not shown). As a result, “unknown” race in BioVU should not be used as an indicator of minority population status—it is far more likely that individuals with “unknown” race are of European-descent.

In conclusion, administratively assigned race is an accurate predictor of genetic ancestry for the ascertainment of European-descent and African-descent individuals, but is less accurate for other diverse populations. Investigators accessing Asian-descent or Hispanic-descent populations should expect a moderate number of samples to have administrative race labels inconsistent with genetic ancestry. When race is an important factor in a study, we recommend, when possible, that a low-cost genotyping array, such as a fixed content Illumina BeadChip (i.e. Illumina HumanCore array) be used to genotype ancestry-informative markers (AIMs) to determine genetic ethnicity.

## Supporting Information

Table S1 **Race/ethnicity terminology usage.**
(DOC)

Table S2 **Percentages of each administratively-assigned race assigned to each genetic ancestry based on PCA clustering group.**
(DOC)

Table S3 **Kappa agreement statistics, including percent agreement and expected agreement, by genetic ancestry, as determined by clustering.**
(DOC)

Table S4 **Genetic ancestry for samples with administratively-assigned race listed as 'unknown'**.
(DOC)
