# Supplementary material for: Accuracy of Administratively-Assigned Ancestry for Diverse Populations in an Electronic Medical Record-Linked Biobank
Source: PLoS One. 2014 Jun 4;9(6):e99161. doi: 10.1371/journal.pone.0099161 (PMC4045967; doi:10.1371/journal.pone.0099161)
Supplement: Table S1 — Race/ethnicity terminology usage. (DOC) [file pone.0099161.s001.doc]

**Table S1**. Race/ethnicity terminology usage.

| **BioVU Administrative Assigned Race Codes** | **Common Use of Administrative Racial Code*** | **Corresponding HapMap Population(s)** | **Genetic Ancestry Defined by PCA Clustering** |
| --- | --- | --- | --- |
| White (W) | Caucasian | CEU | European-descent |
| Black (B) | African American | YRI | African-descent |
| Asian/Pacific (A) | Asian/Pacific | JPT/CHB | East Asian-descent |
| Hispanic (H) | Hispanic | MXL | Hispanic-descent |
| Native American (N) | Native American, Indian, or Other | - | - |
| Indian (I) |  | GIH | South Asian-descent |
| Unknown (U) | Unknown | - | - |

*According to clinical habits, administrative racial codes N and I are often used interchangeably and sometimes used to represent “other” ethnicities
