# Supplementary material for: Accuracy of Administratively-Assigned Ancestry for Diverse Populations in an Electronic Medical Record-Linked Biobank
Source: PLoS One. 2014 Jun 4;9(6):e99161. doi: 10.1371/journal.pone.0099161 (PMC4045967; doi:10.1371/journal.pone.0099161)
Supplement: Table S2 — Percentages of each administratively-assigned race assigned to each genetic ancestry based on PCA clustering group. (DOC) [file pone.0099161.s002.doc]

**Table S2. Percentages of each administratively-assigned race assigned to each genetic ancestry based on PCA clustering group.**

|  |  | **Genetic Ancestry** | | | | |
| --- | --- | --- | --- | --- | --- | --- |
|  |  | European | African | East Asian | Hispanic | South Asian |
| **Administratively-Assigned Race** | Caucasian | 4,174  (98.6%) | 24  (0.6%) | 8  (0.2%) | 16  (0.4%) | 10  (0.2%) |
|  | African American | 11  (1.0%) | 1,080  (98.7%) | 0  (0.0%) | 3  (0.3%) | 0  (0.0%) |
|  | Asian/Pacific | 9  (3.9%) | 0  (0.0%) | 182  (79.8%) | 2  (0.9%) | 35  (15.4%) |
|  | Hispanic | 58  (25.2%) | 8  (3.5%) | 2  (0.9%) | 154  (67.0%) | 8  (3.5%) |
|  | Native American | 90  (48.9%) | 17  (9.2%) | 18  (9.8%) | 18  (9.8%) | 41  (22.3%) |
|  | Indian | 3  (42.9%) | 2  (28.6%) | 0  (0.0%) | 0  (0.0%) | 2  (28.6%) |
|  | Unknown | 1,126  (88.2%) | 83  (6.5%) | 26  (2.0%) | 21  (1.6%) | 21  (1.6%) |
| **HapMap Samples** | CEU | 165  (100.0%) | 0  (0.0%) | 0  (0.0%) | 0  (0.0%) | 0  (0.0%) |
|  | YRI | 0  (0.0%) | 203  (100.0%) | 0  (0.0%) | 0  (0.0%) | 0  (0.0%) |
|  | JPT | 0  (0.0%) | 0  (0.0%) | 113  (100.0%) | 0  (0.0%) | 0  (0.0%) |
|  | CHB | 0  (0.0%) | 0  (0.0%) | 137  (100.0%) | 0  (0.0%) | 0  (0.0%) |
|  | MXL | 5  (5.8%) | 0  (0.0%) | 0  (0.0%) | 75  (87.2%) | 6  (7.0%) |
|  | GIH | 2  (2.0%) | 0  (0.0%) | 0  (0.0%) | 0  (0.0%) | 99  (98.0%) |

Sample counts as well as percentages within each assigned race or HapMap group are shown. Shading ranges from red to green, with high/perfect agreement colored as red, meaning that for a given assigned race or HapMap group most/all of the samples were assigned to the ‘correct’ corresponding cluster.
