# Supplementary material for: Accuracy of Administratively-Assigned Ancestry for Diverse Populations in an Electronic Medical Record-Linked Biobank
Source: PLoS One. 2014 Jun 4;9(6):e99161. doi: 10.1371/journal.pone.0099161 (PMC4045967; doi:10.1371/journal.pone.0099161)
Supplement: Table S3 — Kappa agreement statistics, including percent agreement and expected agreement, by genetic ancestry, as determined by clustering. (DOC) [file pone.0099161.s003.doc]

**Table S3. Kappa agreement statistics, including percent agreement and expected agreement, by genetic ancestry, as determined by clustering.**

|  | *Overall* | | | | *Male* | | | | *Female* | | | |
| --- | --- | --- | --- | --- | --- | --- | --- | --- | --- | --- | --- | --- |
| **Genetic Ancestry** | **Kappa** | **SE** | **Agree** | **Exp.** | **Kappa** | **SE** | **Agree** | **Exp.** | **Kappa** | **SE** | **Agree** | **Exp.** |
| Overall | 0.872 | 0.009 | 94.3 | 55.4 | 0.862 | 0.015 | 94.7 | 61.3 | 0.876 | 0.012 | 94.0 | 51.5 |
| European-descent | 0.906 | 0.013 | 96.2 | 59.5 | 0.906 | 0.020 | 96.6 | 64.2 | 0.904 | 0.017 | 95.8 | 56.7 |
| African-descent | 0.964 | 0.013 | 98.9 | 69.7 | 0.970 | 0.020 | 99.2 | 74.4 | 0.960 | 0.017 | 98.7 | 66.6 |
| East Asian-descent | 0.825 | 0.013 | 98.8 | 92.9 | 0.800 | 0.020 | 98.9 | 94.6 | 0.836 | 0.017 | 98.7 | 91.8 |
| Hispanic-descent | 0.718 | 0.013 | 98.1 | 93.2 | 0.683 | 0.020 | 98.1 | 94.1 | 0.738 | 0.017 | 98.1 | 92.5 |
| South Asian-descent | 0.284 | 0.012 | 96.6 | 95.3 | 0.237 | 0.018 | 96.4 | 95.3 | 0.318 | 0.016 | 96.8 | 95.3 |

Abbreviations: Standard error (SE), percent agreement (Agree), percent expected by chance (Exp). Unknown samples were excluded.
