# Supplementary material for: Accuracy of Administratively-Assigned Ancestry for Diverse Populations in an Electronic Medical Record-Linked Biobank
Source: PLoS One. 2014 Jun 4;9(6):e99161. doi: 10.1371/journal.pone.0099161 (PMC4045967; doi:10.1371/journal.pone.0099161)
Supplement: Table S4 — Genetic ancestry for samples with administratively-assigned race listed as 'unknown'. (DOC) [file pone.0099161.s004.doc]

**Table S4. Genetic ancestry for samples with administratively-assigned race listed as 'unknown'.**

| **Genetic Ancestry** | **Overall** | **Males** | **Females** |
| --- | --- | --- | --- |
| European-descent | 1,126 (88.2%) | 489 (86.5%) | 637 (89.5%) |
| African-descent | 83 (6.5) | 38 (6.7) | 45 (1.4) |
| East Asian-descent | 26 (2.1) | 16 (2.8) | 10 (6.3) |
| Hispanic-descent | 21 (1.6) | 11 (2.0) | 10 (1.4) |
| South Asian-descent | 21 (1.6) | 11 (2.0) | 10 (1.4) |
| **Total** | **1,277 (100)** | **565 (100)** | **712 (100)** |

The samples listed as unknown in BioVU, for our dataset, are 44.2% male and 55.8% female.
